# Codified Medical Law of New York State

**Published:** 1887-08

**Authors:** 


### Selections.

### *CODIFIED MEDICAL LAW OF NEW YORK STATE.*

The following will doubtless be of interest to our readers:

CHAPTER 647.

An Act to regulate the licensing and registration of physicians and surgeons, and to codify the medical laws of the State of New York.

Passed June 23, 1887; three-fifths being present.

*The People of the State of New York, represented in Senate and Assembly, do enact as follows:*

SECTION 1. No person shall practice physic or surgery in this State who shall not have attained the age of twenty-one years; and no person shall practice as aforesaid unless he or she shall be, at the time this act shall take effect, a person lawfully engaged in such practice in this State under license or authority conferred by its laws then in force, and lawfully registered pursuant to chapter five hundred and thirteen of the laws of eighteen hundred and eighty, and the acts amendatory thereof, or unless he or she shall be licensed or authorized so to practice by the provisions of this act, and registered as herein prescribed.

SEC. 2. From and after the date of the taking effect of this act, no person not theretofore licensed or authorized to practice physic or surgery in this State shall be deemed so licensed or authorized except one of the three following classes :

First. All who shall have been graduated from an incorporated medical school or college in this State with the degree of doctor of medicine, after substantial compliance with all the requirements of the general laws and of the charter of said corporation regulating the term and amount of study, attendance and attainment requisite to obtain said degree ; provided that no person shall receive the degree of doctor of medicine, or be licensed to practice physic or surgery in this State, unless after the age of eighteen he shall have pursued the study of medical science for at least three years in a chartered medical school or with some physician or surgeon duly authorized by law to practice physic or surgery ; and shall have attended two complete courses of lectures in some legally incorporated medical school or college, in good standing at the time of such attendance, prior to the granting to him or her a diploma or license ; provided, further, that two courses of lectures, both of which shall be either begun or completed within the same calendar year, shall not satisfy the above requirement.

Second. All who have received said degree from the Regents of the University of the State of New York after substantial compliance with the legal requisites preliminary to its attainment, and after examination by a legally constituted board of medical examiners of this State.

Third. All who, having been graduated from incorporated medical schools or colleges without the State as doctors of medicine, or licensed to practice physic or surgery under the laws of those European countries in which said degree does not confer the right so to practice, shall procure their diplomas from said corporations, or their licenses from such countries, to be indorsed by the faculty of an incorporated medical school or college within this State, or by the Regents of the University on the recommendation of a legally constituted board of medical examiners of this State. Every such indorsement shall be in form of Schedule A or of Schedule B provided by the tenth section of this act. Every corporation or board so indorsing, shall keep a record of their indorsements, and may require applicants to verify their statements under oath ; any indorsement made with fraudulent intent, or gross carelessness or ignorance, shall be deemed a misdemeanor and shall subject the indorser or indorsers, upon conviction thereof, to a fine of two hundred and fifty dollars.

SEC. 3. Every person who, at the time this act shall take effect, shall be practicing lawfully physic or surgery in this State, under the authority and license conferred by the laws then in force, but who shall not be then duly registered in the county where he or she practices ; and every person who shall thereafter become lawfully authorized or licensed to practice physic or surgery in this State, shall register in a book to be kept in the clerk's office of the county in which such practice is carried on, his or her name, residence, place and date of birth, and authority for practicing as aforesaid. Every person who shall apply to register as a physician or surgeon shall be required, before registration, to subscribe and verify by oath or affirmation, before a person qualified to administer oaths in this State, an affidavit which shall be filed and preserved in a bound volume. This affidavit shall be in the form prescribed in

Schedule C, provided by the tenth section of this act. Every person registering as aforesaid shall exhibit to the county clerk his or her diploma or license, or in case of loss, a copy of either, legally certified as are copies of documents admitted in evidence, or a duly attested transcript of the record of its conferment from the body conferring it, upon which the said clerk shall indorse or stamp his name, and the words " Presented and registered as authority to practice physic and surgery by , on the day of , in the clerk's office of county." The said clerk also give to every registered physician or surgeon a certificate in the form of Schedule D, provided by the tenth section of this act. For all of his said services the county clerk shall receive as a total fee for registration, affidavit and certificate the sum of one dollar. It is provided, however, that nothing in this act shall require any physician or surgeon who shall have duly registered lawful authority to practice as such, conformably to the provisions of chapter five hundred and thirteen of the laws of eighteen hundred and eighty, and the acts amendatory thereof, to register again under the provisions of this act, in any county where he or she shall have registered already.

SEC. 4. A practicing physician or surgeon having registered lawful authority to practice physic or surgery in one county, who shall remove his practice or part thereof to or regularly engage in practice or open an office in another county, shall exhibit in person to the clerk of such other county, or shall send to him through the mail by registered letter, his certificate of registration, and if such certificate shows lawful authority to have been registered, said clerk thereupon shall register said applicant in said latter county, on receipt of a fee of twenty-five cents. The clerk shall stamp or indorse upon such certificate the words " Registered also in county," and return the same, and every certificate and indorsement made pursuant to the provisions of this act shall be prima facie evidence in any legal proceeding that the person named has registered in the office issuing the same, the authority stated in the transcript.

SEC. 5. Every person now licensed to practice physic or surgery in this State under the laws thereof in force at the time of the conferment of such license, unless he or she already shall have registered his or her name, residence, place of birth and authority so to practice pursuant to the provisions of section two of chapter five hundred and thirteen of the laws of eighteen hundred and eighty, and the acts amendatory thereof, shall comply with said requirements of said chapter on or before the first day of October, eighteen hundred and eighty-seven; and thereafter no person shall be entitled to register any authority to practice physic or surgery, except the license conferred under this act, and the laws enacted hereafter, and no registration shall be considered valid as such unless the authority registered constituted at the time of registration a license under the laws of this State then in force; provided that nothing in this section shall be construed to prohibit or suspend any prosecution for non-registration under said section instituted prior to said first day of October, eighteen hundred and eighty-seven, and further provided, that no diploma or license conferred upon a person not actually in attendance at the lectures, instruction and examination of the corporation conferring the same, or not possessed at the time of its conferment of the requirements then demanded of medical students in this State as a condition of their becoming licensed so to practice, shall be deemed lawful authority to practice physic and surgery in this State.

Sec. 6. No person shall be licensed or permitted to practice physic or surgery in this State who has been convicted of a felony by any court of competent jurisdiction; and if any person who is or hereafter shall be duly licensed to practice physic or surgery in this State, shall be convicted of a felony, as aforesaid, his or her license to so practice, if any, shall be revoked by the fact of such conviction having been had. Any person who shall willfully swear falsely to any statement contained in any affidavit made pursuant to the provisions of this act, shall be deemed guilty of a felony, and subject to conviction and punishment for perjury; any person who falsely and without authority shall counterfeit, make or alter any diploma, certificate or instrument constituting a license to practice physic or surgery within this State, or any certificate or indorsement given in pursuance of this act, shall be deemed guilty of a felony, and be subject to conviction and punishment for forgery in the second degree; any person who shall practice physic or surgery under a false or assumed name, or who shall falsely personate another practitioner of a like or different name, shall be deemed guilty of a felony, and shall be subject to conviction and punishment for false personation; and any person guilty of violating any of the other provisions of this act, not otherwise specifically punished herein, or who shall buy, sell or fraudulently obtain any medical diploma, license, record or registration, or who shall aid or abet such buying, selling or fraudulently obtaining thereof, or who shall practice physic or surgery in this State under cover of a diploma or license that shall have been illegally obtained, or that shall have been signed or issued unlawfully or under fraudulent representations, or mistake of fact in material regard, or who, after conviction of a felony, as aforesaid, shall attempt to practice physic or surgery in this State, and any person who shall assume the title of doctor of medicine, or append the letters "M. D." to his or her name, without having received the degree of doctor of medicine from some school, college or board empowered by law to confer said degree or title, shall be deemed guilty of a misdemeanor, and upon conviction thereof shall be punished by a fine of not less than two hundred and fifty dollars, or imprisonment for six months for the first offense, and upon conviction of a subsequent offense, by a fine of not less than five hundred dollars or imprisonment for not less than one year, or by both fine and imprisonment. Any person who, not being then lawfully authorized to practice physic or surgery in this State and so registered according to law, shall practice, on or after the first day of October, eighteen hundred and eighty-seven, physic or surgery within this State without the license and registration provided for in this act, shall be deemed guilty of a misdemeanor, and on conviction thereof shall be punished by a fine of not less than fifty dollars for the first offense, and for each subsequent offense by a fine of not less than one hundred dollars, or by imprisonment for not less than one hundred days, or by both fine and imprisonment. When any prosecution under this act is made on the complaint of a lawfully incorporated medical society of this State, or a county society entitled to representation in a State society or association, the fines when collected shall be paid to the society making the complaint, and any excess of the amount of fines so paid over the expense incurred by the said society in enforcing the medical law of this State, shall be paid at the end of the year to the county treasurer, for the use of the poor of said county.

SEC. 7. The duly incorporated medical societies of any county in which any person shall practice physic or surgery without lawful authority or registration may, upon proof of such practice, recover from such practitioner, in an action before any justice of the peace, a penalty of twenty-five dollars and the cost of the action for the first judgment, and upon every subsequent judgment for the same offense a penalty of fifty dollars and the cost of the action; provided that said societies shall pay to the county treasurer for the use of the poor of said county any surplus that may accrue in their hands from the excess of fines and penalties collected over the disbursements of said society for counsel fees and the expenses incident to the enforcement of this act by them.

SEC. 8. Nothing in this act shall be construed to punish commissioned medical officers serving in the army or navy of the United States, or in the United States marine hospital service, while so commissioned, or any one while actually serving as a member of the resident medical staff of any legally incorporated hospital, or any legally qualified and registered dentist exclusively engaged in practicing the art of dentistry, or interfere with manufacturers of artificial eyes, limbs or orthopedical instruments or trusses of any kind from fitting such instruments on persons in need thereof; or any lawfully qualified physicians and surgeons residing in other States or countries meeting registered physicians and surgeons of this State in consultation, or any physician or surgeon residing on the border of a neighboring State, and duly authorized under the laws thereof to practice physic or surgery therein, whose practice extends into the limits of this State; providing that such practitioner shall not open an office or appoint a place to meet patients or receive calls within the limits of the State of New York; or physicians duly registered in one county of this State, called to attend isolated cases in another county, but not residing or habitually practicing therein.

SEC. 9. The following acts and parts of acts are hereby expressly repealed, to-wit: Sections eight to twenty-two inclusive of title seven of chapter fourteen of part one of the Revised Statutes; also all of chapter one hundred and thirty-eight of the laws of eighteen hundred and six, that provided for the examination and admission of medical students to practice, and for penalties for practicing physic and surgery without a diploma or other lawful authority; also section or paragraph fifth of chapter one hundred and four of the laws of eighteen hundred and seven; also sections nine, ten, eighteen and all of section eleven following and including the words "whose duty," of chapter ninety-four of the laws of eighteen hundred and thirteen; also sections one, two, four, and all of section three following and including the words "any three," of chapter two hundred and six of the laws of eighteen hundred and eighteen; also section two of chapter two hundred and thirty-seven of the laws of eighteen hundred and nineteen; also chapter one hundred and twenty-six of the laws of eighteen hundred and thirty; also sections one, two and four of chapter five hundred and thirty-two of the laws of eighteen hundred and thirty-six; also chapter sixty-four of the laws of eighteen hundred and forty-one; also chapter two hundred and seventy-five of the laws of eighteen hundred and forty-four; also chapter four hundred and thirty-six of the laws of eighteen hundred and seventy-four; also chapter five hundred and thirteen of the laws of eighteen hundred and eighty; also chapter one hundred and eighty-six of the laws of eighteen hundred and eighty-one; also chapters four hundred and eleven and four hundred and forty-five of the laws of eighteen hundred and eighty-four; also section three hundred and fifty-six of the Penal Code. And also all acts or parts of acts authorizing any incorporated school or college to confer the degree of doctor of medicine *causa honoris* or *ad eundem*, or otherwise, than upon duly graduated students in course; and all other acts or parts of acts inconsistent with this act are hereby repealed. And it is provided that the degree of doctor of medicine conferred *causa honoris* or *ad eundem gradum*, shall not be a qualification for the practice of physic and surgery in this State. And, whereas, it is the purpose of this act to codify the statutory provisions of this State regulating the admission of individuals to the practice of physic and surgery, and the punishment of those practicing either physic or surgery without authority. It is further provided that the specific repeal herein of any portion of an act that may have been heretofore repealed, expressly or by implication, shall not be construed to revive the remaining part thereof.

SEC. 10. Section ten shall embrace the following schedules, namely A, B, C and D. * * *

[Here follows forms for registration and for the endorsement of diplomas.]

